# An Exploratory Analysis of Genetic Structure and Introgression in *Salmo trutta* Populations from Campania Rivers

**DOI:** 10.3390/ani16193061

**Published:** 2026-09-29

**Authors:** Angela Michela Immacolata Montone, Andrea Fulgione, Anna Cutarelli, Alberto Gentile, Roberta Brunetti, Francesco Paolo Serpe

**Affiliations:** 1 Department of Food Security Coordination, Istituto Zooprofilattico Sperimentale del Mezzogiorno, 80055 Portici, NA, Italy; anna.cutarelli@izsmportici.it (A.C.); francesco.serpe@izsmportici.it (F.P.S.); 2 Department of Agricultural Sciences, University of Naples Federico II, 80055 Portici, NA, Italy; 3 OMNIA Ecosystem Srl., 84086 Roccapiemonte, SA, Italy; gentile.alberto79@gmail.com; 4 Epidemiology and Biostatistics Coordination Department, Istituto Zooprofilattico Sperimentale del Mezzogiorno, 80055 Portici, NA, Italy; roberta.brunetti@izsmportici.it

**Keywords:** Campania region, genetic introgression, LDH-C1*, microsatellite, *Salmo trutta*

## Abstract

The *Salmo trutta* complex exhibits high genetic and phenotypic diversity due also to the historical climatic events and colonization processes. In Europe, several evolutionary lineages have been identified, including the Atlantic and Mediterranean. In Italy, native Mediterranean trout populations have been threatened by restocking practices involving hatchery-reared fish of Atlantic origin, thus resulting in widespread hybridization and genetic introgression. This study aimed to genetically characterize trout populations from selected river basins of the Campania region (southern Italy) and to assess whether the observed morphological characteristics, consistent with standard morphological descriptions of Mediterranean trout, were supported by genetic evidence (i.e., LDH-C1* locus and microsatellite markers analyses). The results revealed a complex genetic structure of *Salmo trutta* populations in Campania, with evidence of Atlantic introgression in several river systems. Differences between LDH-C1* genotyping and microsatellite data highlighted the importance of using multiple genetic markers to accurately detect hybridization and identify lineage composition. These explorative results have also evidenced that specific populations (i.e., those from Imperatore, Sava, and Volturno rivers), showed genetic features consistent with Mediterranean ancestry. The findings highlight the importance of genetic analyses not only for accurate species identification beyond morphology but also for supporting conservation strategies to protect native Mediterranean trout and guide restocking based on local genetic resources.

## Simple Summary

In Italy, the native Mediterranean trout populations are threatened by the introduction of hatchery-reared trout of Atlantic origin, which can cause hybridization and loss of local genetic biodiversity. This study aimed to provide a preliminary genetic characterization of the trout populations in selected river basins of Campania region (southern Italy), and to determine whether morphological differences between Atlantic and Mediterranean trout are also supported by the genetic analyses of the LDH-C1* gene and microsatellite markers. The results showed a complex genetic structure among Campanian trout populations, with evidence of Atlantic genetic introgression in different rivers; and that the analysis of multiple genetic markers is important for accurately identifying hybridization and assessing trout ancestry. However, trout specimens from rivers such as Imperatore, Sava, and Volturno showed genetic characteristics consistent with Mediterranean ancestry. These findings can contribute to the conservation of native Mediterranean trout, the preservation of local genetic diversity, and the development of more effective restocking strategies based on genetically appropriate local populations.

## 1. Introduction

The family Salmonidae includes three subfamilies: whitefish (*Coregoninae*), grayling (*Thymallinae*) and salmonids (*Salmonidae*). This last consists of the genus *Salmo*, *Brachymystax*, *Salmothymus*, *Acantholinqua*, *Hucho*, *Salvelinus* and *Oncorhynchus* [1]. The genus *Salmo* (*S.*) Linnaeus, 1758 is grouped into only two species: *Salmo salar* and *Salmo trutta* (*S. trutta*); the latter includes most of the European brown trout [2]. *S. trutta* originated from an ancestral form in the Palearctic region and presents different evolutionary lineages. The *S. trutta* complex consists of several geographically and evolutionarily differentiated groups, including Atlantic (ATL) and Mediterranean (MED) lineages, whose genetic profiles can be assessed using molecular markers conventionally employed to characterize Atlantic- and Mediterranean-associated genetic variation [3]. The *S. trutta* has a broad geographic distribution across Europe and adjacent regions, occupying a wide range of freshwater habitats. In Italy, brown trout populations occur across suitable riverine and lacustrine environments from northern to southern regions, with substantial geographic and genetic variation among populations [2,3]. The different lineages of *S. trutta* were principally due to the glaciation processes and specific colonization histories [4]. In 2014, the Italian Association of Fresh Water Ichthyologists (AIIAD) suggested a classification of Italian trout population into evolutionarily significant units (ESU), reflecting their evolutionary history and genetic differentiation. These include *S. marmoratus* in Po valley; *S. cettii* (previously known as *S. macrostigma*) in Sardinia and Sicily, in lakes such as Posta and Fibreno and in some Tyrrhenian waterways; *S. ghigii* (also known as Mediterranean brown trout) presents in Apennine and Tyrrhenian basins; *S. Fibreni* presents in both lakes Posta and Fibreno; and *S. carpio*, located in lake Garda. It is important to highlight that lake trout is not considered a separate species but rather an ecotype of both *S. trutta* and *S. marmoratus* [5]. Nonetheless, the restocking activities and the introduction of allochthonous genotypes into habitats previously occupied by native populations have led to an extensive genetic introgression, resulting in the reduction or—in some cases—the loss of distinct indigenous genetic lineages [6,7,8,9]. Most of the hatchery brown trout used for restocking originate from a Central European stock belonging to the Atlantic lineage, which is genetically distinct from the native populations [10]. From many years, the research has been focused on the status and distribution of native *S. trutta* populations in Italy, primarily for conservation purposes. Several studies have revealed the introgression of trout domesticated populations of northern European origin (i.e., ATL lineage) into the local population such as the Mediterranean (MED lineage) for satisfying the fishing demands and supporting recreational activities [11,12,13,14]. This admixture has further complicated the identification and classification of local *S. trutta* population [15,16]. Although morphological traits (such as the number of parr marks, number of black opercular spots, etc.) can provide a rapid preliminary assessment of trout lineage, their correspondence with genetic ancestry may be affected by hybridization or introgression [17,18]. Therefore, several studies have adopted nuclear *lactate dehydrogenase-C1* (LDH-C1*) *locus*, mitochondrial genes and microsatellite markers to discriminate between Mediterranean and Atlantic trout lineages [13,19,20,21].

In Campania, the relationship between Mediterranean-like morphological traits and genetic background, as well as the extent of Atlantic introgression and the genetic structure of resident trout populations, remains poorly characterized. This study aims to provide a preliminary characterization of *S. trutta* populations inhabiting selected river basins of Campania and to investigate the relationship between their morphological characteristics and genetic profile using the nuclear LDH-C1* locus and microsatellite markers. The nuclear locus LDH-C1* was selected as a widely used lineage-associated genetic marker in brown trout (*Salmo trutta*) population genetics [22,23]. The use of this *locus* provides a reliable screening tool to evaluate population genetic structure and the potential impact of introgressive hybridization from non-native specimens. Furthermore, the microsatellite markers were additionally adopted to provide a more comprehensive assessment of genetic profile.

Integrating genetic information may support the identification and conservation of native trout populations and inform conservation-oriented restocking and management strategies, thereby contributing to the preservation of their genetic background.

## 2. Materials and Methods

### 2.1. Study Area

**Figure 1:**
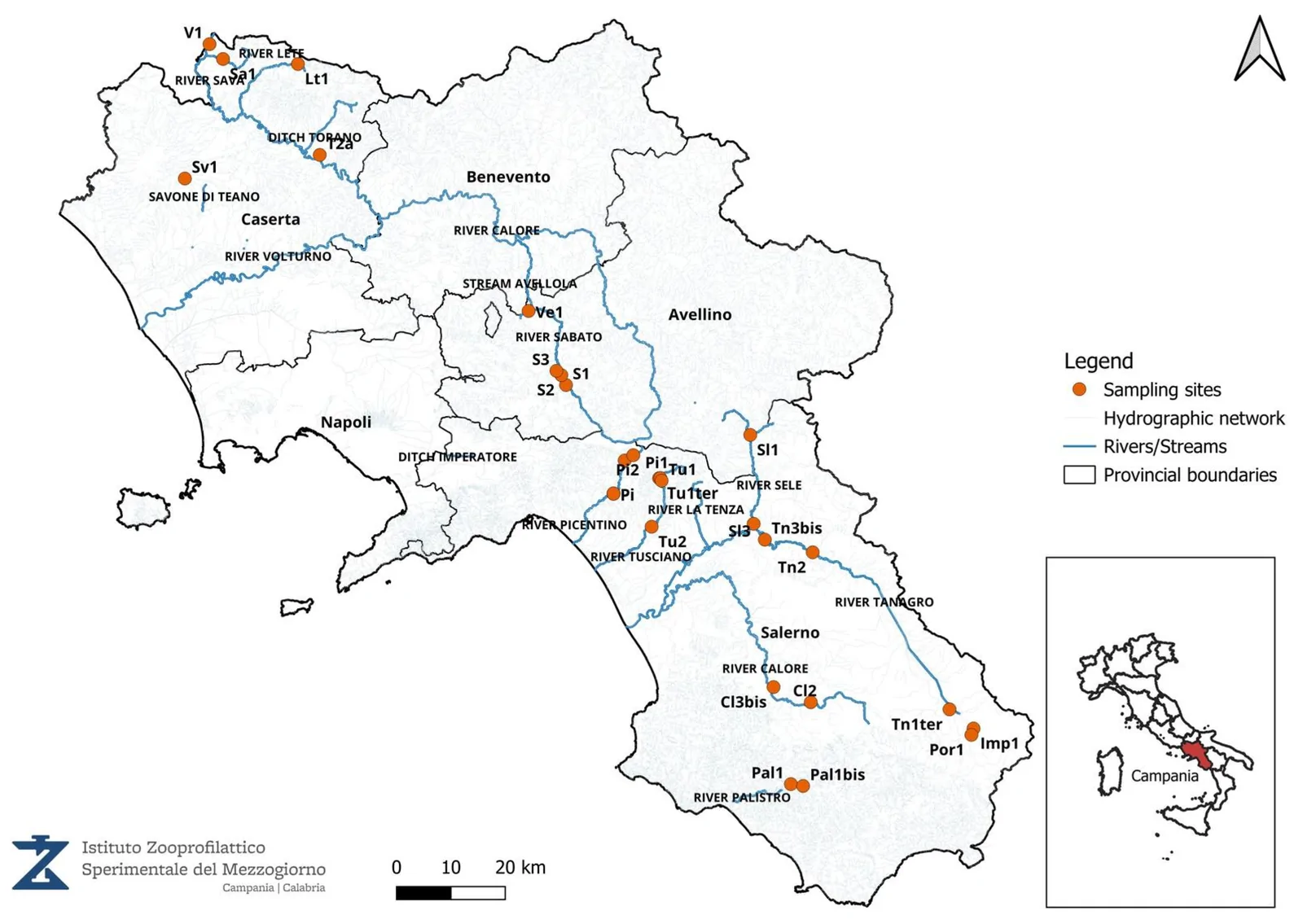
Geographical distribution of the sampling sites in the Campania region (Southern Italy). Orange circles indicate the sampling stations, while blue lines represent the rivers and streams included in the study. The light-blue background network represents the regional hydrographic network, and black lines indicate provincial boundaries. The inset map shows the location of the Campania region.

A total of 596 salmonids were collected from 29 sampling stations located across 16 river stretches (Table 1). Sampling sites were selected according to historical provincial fish classification schemes and geomorphological characteristics of the watercourses, with particular emphasis on habitat conditions known to be suitable for salmonid populations. Their geographic positions were plotted by Osservatorio Regionale Sicurezza Alimentare (ORSA) of the Istituto Zooprofilattico Sperimentale del Mezzogiorno by means of the software QGIS 3.22 http://qgis.org (Figure 1).

### 2.2. Trout Specimens Collection

Salmonid specimens were collected by electrofishing and using the multiple-pass depletion method [17,24,25,26,27]. By this way, each river stretch was sampled at least twice, progressing from downstream to upstream under standardized effort conditions [28]. For each specimen, phenotypic traits including the presence of any diseases or malformations were recorded for estimating population structure. On-site photographs were taken of each specimen with the dorsal fin fully extended to facilitate the accurate identification of morphological reference landmarks. Furthermore, a small tissue sample (e.g., adipose fin fragment) was collected from each specimen and stored in 2 mL tubes containing absolute ethanol ≥ 99.8% (Sigma-Aldrich, Merck KGaA, Saint Louis, MO, USA) for the following genetic analyses. Each sample was labelled with a unique alphanumeric code indicating the sampling station and specimen number. After the collection, all trout specimens were immediately released alive at their capture site by trained personnel. The study was conducted under the PO FEAMP 2014–2020, Measure 1.44, and in accordance with Italian legislation (DL n. 26, 4 March 2014, implementing Directive 2010/63/EU) [29,30]. Since the study involved no experimental manipulation or procedures causing pain, suffering, distress, or lasting harm to the animals, no additional Animal Ethics Committee approval was required.

### 2.3. Phenotypic Characterization

Each specimen was initially classified by a direct visual inspection, and its external morphological and pigmentation traits were recorded descriptively. Particular attention was given to the presence, size, and definition of preopercular and opercular spots, as well as to overall flank and dorsal coloration, vermiculation, the distribution and pigmentation of body speckles and spots, the presence of pale halos around red spots, and the pigmentation pattern of the fins. General body conformation, including body robustness, head size, and fin development, was also recorded. These observations documented phenotypic variation but were not used to assign individuals to evolutionary lineages.

### 2.4. DNA Extraction, LDH-C1* e 16S rRNA Genotyping

The genomic DNA was extracted and purified from adipose fin fragments using the QIAMP DNA Mini KIT (Qiagen, Milan, Italy). The genetic diversity in trout populations was assessed using nuclear markers (LDH-C1* and 12 microsatellites). Furthermore, the mitochondrial *16S rRNA* gene was analyzed in all trout classified as Mediterranean by LDH-C1* analysis, to independently assess their mitochondrial lineage. The LDH-C1* and *16S rRNA* were both analyzed by Restriction Fragment Length Polymorphism—PCR (RFLP-PCR). The reactions were carried out in a total volume of 25 µL, including 1 µL of DNA at 20 ng/µL, 12.5 µL of Master Mix (EconoTaq PLUS 2X Master Mix, LGC, Teddington, UK), 0.25 µL of each primer (100 µM), and 11 µL of RNase-free water (Thermo Fisher Scientific, Monza, Italy). The nuclear gene LDH-C1* was amplified by using the primers Ldhxon3F (5′-GGCAGCCTCTTCCTCAAAACGCCCAA-3′) and Ldhxon4R (5′-CAACCTGCTCTCTCCCTCCTGCTGACGAA-3′) [16]. PCR products were then digested with BslI to distinguish homozygous and heterozygous genotypes. Mediterranean individuals (*100/100) yielded a 440 bp fragment; Atlantic homozygotes (*90/90) produced two fragments (360 bp and 80 bp); putatively hybrid (*90/100) showed three fragments (440 bp, 360 bp, and 80 bp).

The *16S rRNA* gene was amplified using the universal primers Sar (5′-CGCCTGTTTATCAAAAACAT-3′) and Sbr (5′-CCGGTCTGAACTCAGATCACGT-3′) according to Patarnello et al. [31]. The amplified PCR products were digested using AfaI (RsaI), producing distinct fragment patterns that enable the clear discrimination between Atlantic (360 bp and 80 bp) and Mediterranean haplotypes (a single 440 bp fragment) [2]. RFLP-PCR fragments were separated on a QIAxcel Advanced capillary electrophoresis system (Qiagen, Milan, Italy).

### 2.5. Microsatellites Genotyping

DNA from 120 randomly selected brown trout individuals—with a minimum of two individuals from each of the 29 sampling stations—were genotyped at twelve autosomal microsatellite *loci* using four multiplex PCR sets: the first including *SsaD190*, *SsaD71*, and *SSsp2213*; the second, *Ssa85* and *Ssa410Uos*; the third, *Str73INRA*, *Str60INRA*, *SsoSL417*, and *Ssa408Uos*; and the fourth, *Str15INRA*, *Strutta58*, and *T3-13* (Table 2). Multiplex PCRs were performed using the Qiagen Multiplex PCR Kit (Qiagen, Milan, Italy). PCR reactions were in a total volume of 10 µL, including 3 µL of template DNA at 20 ng/µL, 5 µL of Master Mix (Qiagen, Milan, Italy), 1 µL of Q-Solution (Qiagen, Milan, Italy), and 1 µL of 2.5 µM primer mix. The thermal protocol was 94 °C for 15 min, followed by 45 cycles at 94 °C for 30 s, 57 °C for 90 s, and 72 °C for 60 s, and a final extension at 60 °C for 30 min. The amplified fragments were then mixed with GeneScan 500 LIZ size standard (Thermo Fisher Scientific, Monza, Italy) and Hi-Di Formamide (Applied Biosystem, Monza, Italy), denatured at 95 °C for 3 min, and analysed by using SeqStudio Genetic Analyzer (Applied Biosystems, Monza, Italy). The allele sizes were determined by GeneMapper Software v6 (Applied Biosystems, Monza, Italy). Population structure and individual ancestry were assessed using the software Structure vers. 2.3.4 [32], which estimates both the proportion and genomic assignment of each trout to the inferred genetic clusters [33]. The above software is based on a Bayesian approach, and it uses a Monte-Carlo Markov-Chain (MCMC) algorithm to attribute individuals to a specific genetic cluster “K”. The analyses employed the admixture model with both correlated and uncorrelated allelic frequencies among populations. MCMCs were run for 500,000 generations after 200,000 generations of burn-in. Each value of K, which indicates the number of genetic clusters and ranges from 1 to 8, was tested in five independent replicates. To define the optimal number of K, both the highest log probability of K (LnP method) and the highest rate of change in LnP between successive K were evaluated using Structure Selector [11,34]. For descriptive individual assignment, a membership coefficient of Q ≥ 0.80 was used as an operational threshold to identify individuals predominantly associated with one inferred genetic component. This threshold is consistent with commonly used assignment criteria in Structure-based population and hybridization studies, but it is important to highlight that the optimal threshold depends on the genetic divergence, number of markers, and analytical objective [35,36].

Genetic structure was analyzed using Wright’s fixation index (*F*_ST_ index), calculated by means of Structure Selector. This value can range from 0 (no genetic differentiation) to 1 (complete differentiation).

### 2.6. Microsatellite Quality Control

A *locus*-level quality-control screening was performed on the microsatellite genotype matrix. For each *locus*, the proportion of missing genotypes, number of observed alleles, observed heterozygosity (Ho), and expected heterozygosity (He) were calculated. Potential null-allele effects were assessed using the Brookfield estimator, r^^B^ = (He − Ho)/(1 + He) [43]. Because the specimens originated from multiple geographically distinct sampling sites, heterozygote deficiencies were interpreted cautiously, as population subdivision may produce a Wahlund effect.

## 3. Results and Discussion

### 3.1. Phenotypic Characterization of Trout Specimens by Site

The morphological examination did not allow a reliable assignment of the sampled specimens to either the Mediterranean (MED) or Atlantic (ATL) lineage. Although several morphological and pigmentation traits traditionally associated with these lineages were observed, their distribution was neither sufficiently consistent nor lineage-specific to support individual classification. The examined trout showed overlapping combinations of characters, including variable preopercular and opercular pigmentation, flank coloration and vermiculation, and the distribution and intensity of black and red body spots. Variation in body robustness, head proportions, and fin pigmentation was also observed, but these traits did not provide a consistent basis for distinguishing the two lineages. All the trout specimens have this morphological overlap, displaying a combination of traits reported for both Mediterranean and Atlantic populations, including a well-defined dark preopercular spot and flank vermiculation, together with a relatively regular distribution of flank spots, including red spots with pale halos, and comparatively uniform fin pigmentation. These observations further support the lack of a consistent morphological basis for distinguishing MED and ATL specimens.

Overall, the morphological appearance of the sampled individuals was considered intermediate or overlapping rather than diagnostically attributable to either lineage. Morphological examination was therefore retained as a descriptive assessment of external phenotype, while lineage assignment was based on subsequent genetic analyses. Accordingly, morphological traits were not used as independent criterion to classify individuals as MED, ATL, or hybrid.

### 3.2. LDH-C1* and 16S rRNA Genotyping

**Figure 2:**
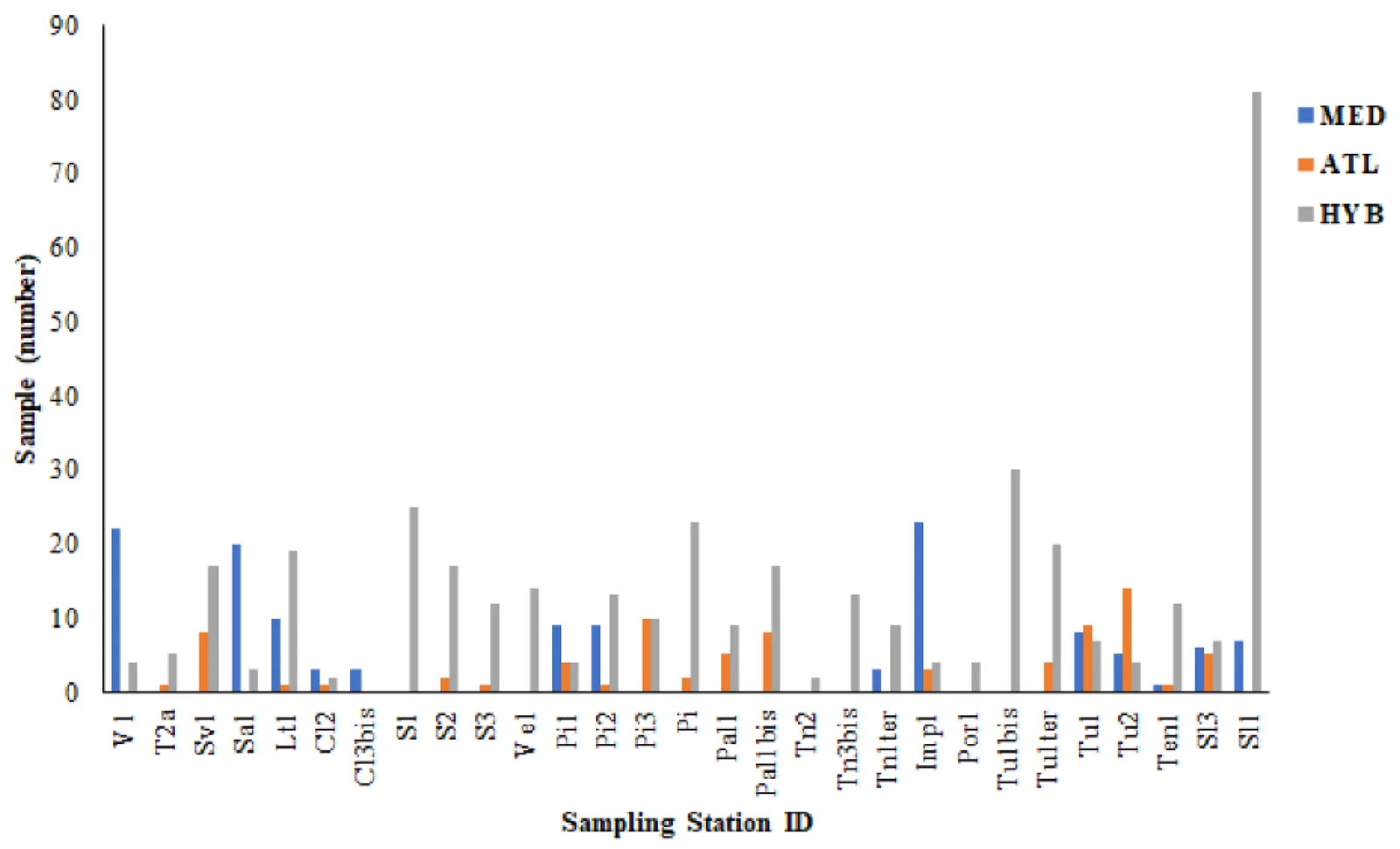
Distribution of Salmo trutta LDH-C1* genotypes. LDH-C1* genotypes of *Salmo trutta* across river sampling sites: Mediterranean (blue), Atlantic (orange), and Hybrid (grey). Number of trout per site (Sampling Station ID) is shown.

The RFLP-PCR analysis of the LDH-C1* gene is widely used in trout phylogeographic and conservation research to assess the introgression degree and the long-term persistence of native genetic lineages [44,45]. In this study, specimens were classified into three genetic groups: Mediterranean (MED), Atlantic (ATL), and putatively hybrid (HYB) (Figure 2). The results evidenced a clear predominance of HYB individuals (387/596; 65%), followed by MED (129/596; 21.6%) and ATL genotypes (80/596; 13.4%). To provide additional support for MED classification, all specimens identified as MED homozygotes (LDH-C1* 100/100) were subsequently analyzed for the mitochondrial *16S rRNA* gene (Table S1). All MED specimens showed a Mediterranean mitochondrial profile, providing concordant evidence from nuclear and mitochondrial markers. However, since *16S rRNA* was not analyzed in the ATL and HYB groups, mito-nuclear discordance could not be assessed across the entire sampled population.

**Figure 3:**
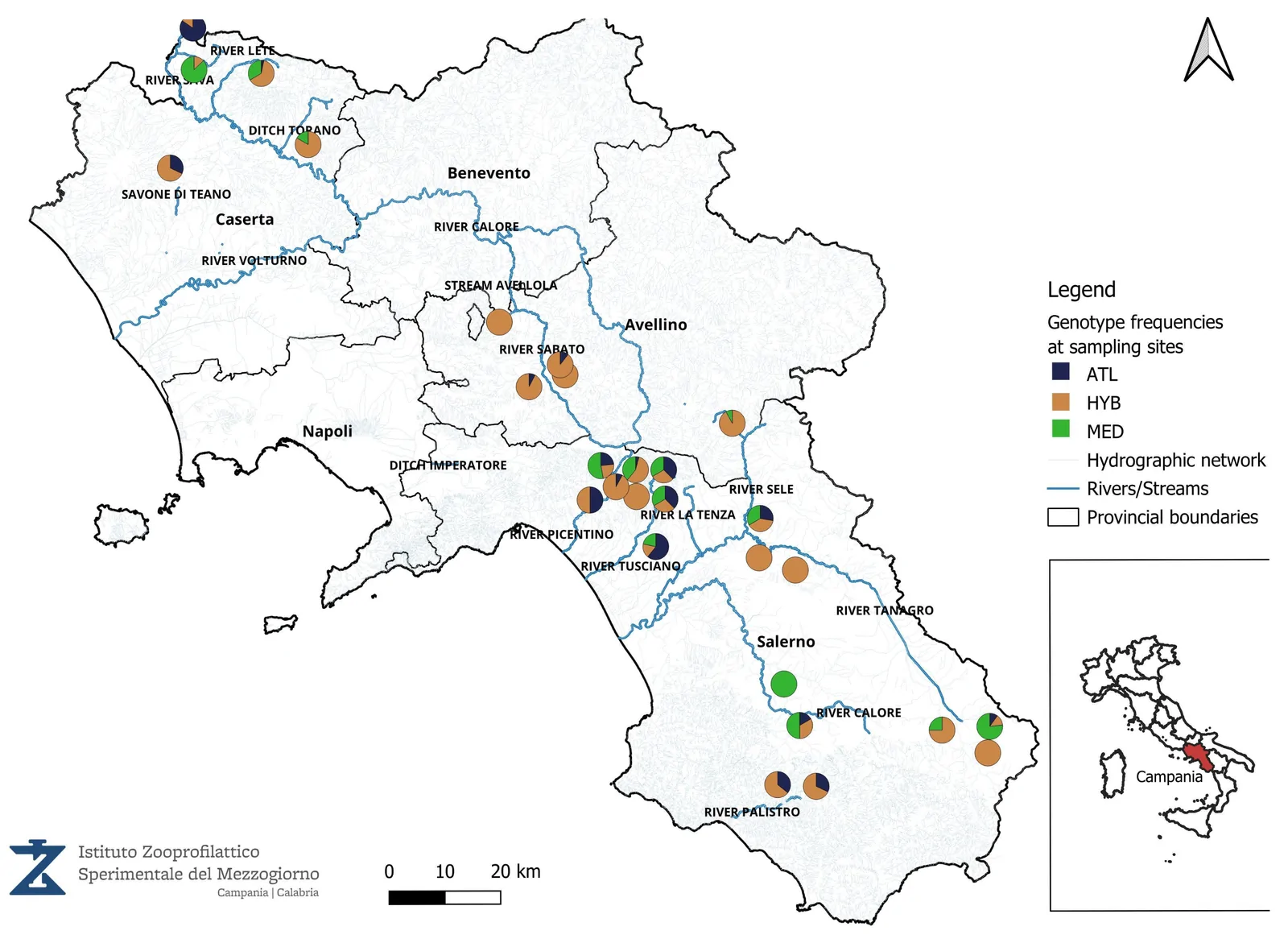
Spatial distribution of LDH-C1* genotypes at the sampling sites in the Campania region (Southern Italy). Pie charts represent the relative frequencies of ATL, HYB, and MED genotypes at each sampling site. Blue lines indicate the rivers and streams included in the study, the light-blue background network represents the regional hydrographic network, and black lines indicate provincial boundaries. The inset map shows the location of the Campania region.

It is important to evidence that LDH-C1* is a widely used nuclear marker for distinguishing Mediterranean-associated from Atlantic/allochthonous genetic components, whereas mitochondrial markers provide information on maternal lineage. Their complementary use can provide a more comprehensive assessment of genetic ancestry, particularly in populations affected by introgressive hybridization, where nuclear and mitochondrial lineages may differ [13,23,46]. Furthermore, the spatial distribution of LDH-C1* genotypes at the sampling sites is shown in Figure 3. The results of LDH-C1* analysis also evidenced a high proportion of the MED-associated genotype at different sampling sites such as Imp1 (Imperatore; 23 individuals), V1 (Volturno; 22 individuals), Sa1 (Sava; 20 individuals), and Cl2 and Cl3bis (Calore; 6 individuals). Specifically, at Imp1 site, the Mediterranean trout accounted for over 76.7% of the total catch (23 out of 30 individuals), thus indicating the persistence of a relatively well-conserved native gene pool. Similarly, high frequencies were also observed at the Volturno (22 out of 26), Sava (20 out of 23), and Calore (6 out of 9) sites. These data allowed us to hypothesize that geographic isolation and/or limited anthropogenic pressure may have contributed to the preservation of the native genetic background in these systems. Sampling sites such as Sl1 (Sele), Tu1ter and Tu1bis (Tusciano), P1 (Picentino), Pal1 and Pal1bis (Palistro), Sv1 (Savone), Tn3bis (Tanagro), Vel1 (Vellola), T2a (Torano) and Por1 (Porcile) were, instead, dominated by the presence of hybrids (HYB) specimen, with frequencies exceeding 80% in some cases. These findings align with areas historically subjected to massive stocking activities and river regulation, where native populations have become genetically swamped [47].

ATL specimens were, instead, moderately represented at sites such as Pi3 (Picentino) and Tu2 (Tusciano), where it commonly co-occurred with the hybrids. The presence of ATL-associated genotype in these areas could be linked to past introduction events, rather than evidence of ongoing admixture, as their distribution showed minimal overlap with that of Mediterranean genotype. The relatively high frequency of MED-associated genotypes in some rivers could be probably due to several ecological and management-related factors. For example, the Imperatore River has not been subjected to restock program with Atlantic brown trout for over two decades, reducing the likelihood of genetic introgression and potentially favoring the persistence of MED-associated genetic variation. The Volturno River, due to its large size and environmental complexity, could pose ecological constraints to the establishment of Atlantic lineage strains, thus probably favoring the persistence of native Mediterranean genotypes. Furthermore, the Sava River—hydrologically connected to Volturno—may act as a natural route for the migration and dispersal of Mediterranean brown trout, potentially facilitating their occurrence in both river systems. Conversely, in rivers where MED-associated genotypes were not highly prevalent, restocking practices and environmental pressures may have contributed to the observed genetic composition. Rivers such as Velolla, Savone, Palistro, Sabato, Porcile, and Torano have been subjected to repeated stocking with Atlantic origin specimens from the Ceraso hatchery, which may have contributed to the introgression of Atlantic-associated genetic variation into native MED populations. Furthermore, some rivers such as Velolla, are characterized by low population densities and environmental stressors, including summer droughts, which may affect the persistence of native populations. In the Palistro basin, anthropogenic pressure may have contributed to changes in local population structure, suggesting that ecological disturbance and intensive management practices may act jointly in shaping population dynamics [48].

The spatial distribution of HYB individuals should not be interpreted as evidence that hybridization occurred at the same sampling site or during the same reproductive period. Hybrid genotypes may persist for several generations after an original inter-lineage cross, may disperse between connected river sections, or may result from historical stocking and subsequent reproduction. Consequently, HYB individuals can occur at sites when one or both parental genotype classes are not currently detected. Conversely, the co-occurrence of MED and ATL individuals indicates the potential for inter-lineage contact but does not demonstrate local hybridization.

### 3.3. Microsatellite Genotyping and Lineage Assignment by Structure

In addition to LDH-C1* and *16S rRNA* analyses, a representative panel of 120 individuals was further genotyped using microsatellite markers. First, a *locus*-level quality-control screening was performed to evaluate missing data, allelic diversity, observed heterozygosity (Ho), expected heterozygosity (He), and potential null-allele effects. *Loci* showing potential null-allele effects were not excluded a priori. Their influence was considered for the interpretation of the multi *locus* Structure results; and *locus* specific differentiation screening was performed to assess whether clustering was disproportionately driven by individual markers. No single *locus* was found to drive the observed subdivision; therefore, all *loci* were retained in the final analysis.

Missing genotypes ranged from 1.7% to 12.5%. Several *loci* showed heterozygote deficiencies: *Str73INRA* (Ho = 0.055; He = 0.726; estimated null-allele frequency = 0.389), *Str60INRA* (Ho = 0.241; He = 0.723; estimated null-allele frequency = 0.280), and *Str15INRA* (Ho = 0.254; He = 0.708; estimated null-allele frequency = 0.266). Intermediate estimates were observed for *Ssa85* (0.241) and *SsoSL417* (0.191), whereas the remaining *loci* showed lower estimated null-allele frequencies (Table S2). These results indicate that *locus*-specific effects, including possible null alleles, should be considered when interpreting the microsatellite-based structure. It is important to highlight that the discrepancies observed by Structure analysis were not attributable to a single problematic *locus*, as several *loci* contributed to the differentiation among inferred components.

**Figure 4:**
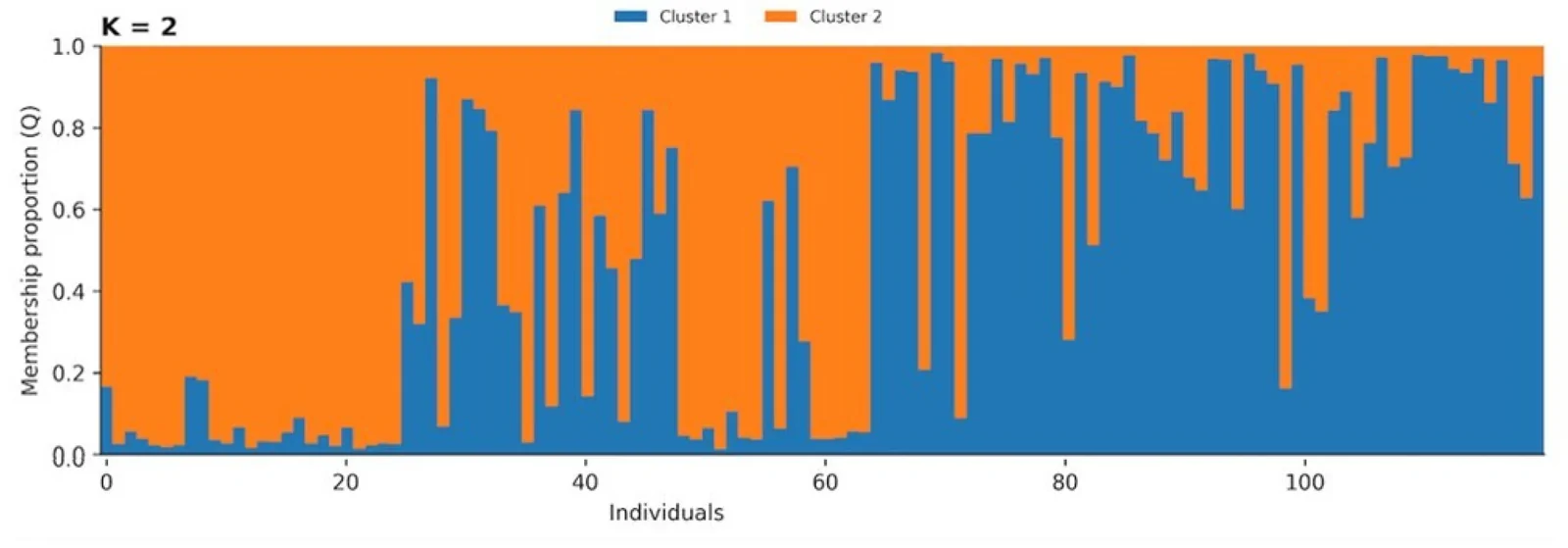
Structure analysis of *Salmo trutta* individuals. Individual ancestry coefficients inferred under K = 2. Each vertical bar represents one individual, with colors indicating the estimated membership proportions (Q-values) in the inferred genetic components. Individuals are shown in the same order in both panels to facilitate direct comparison between the two clustering solutions. Individual identification, sampling site, LDH-C1* genotype, and corresponding Q-values are reported in Table S4.

The K = 2 Structure solution was used as the principal description of the multi *locus* genetic structure (Figure 4). Overall membership coefficients were 0.496 and 0.504 for clusters 1 and 2, respectively. Expected heterozygosity was 0.7951 and 0.8743, while Structure-derived FST estimates were 0.0555 and 0.0004, respectively. The estimated log probability of the data was LnP(D) = −6134.3, with a mean log likelihood of −5927.6, variance of 413.2, and alpha of 0.1477 (Table S3). Using Q ≥ 0.80 as an operational threshold, 85 of 120 individuals (70.8%) showed predominant assignment to one K = 2 component, whereas 35 individuals (29.2%) had Q < 0.80 for both components and were considered admixed for descriptive purposes. Individual Q-values, sampling sites, and cluster assignments are reported in (Table S4).

The value K = 3 was retained as an exploratory comparison rather than as evidence for a third biological lineage. The K = 3 solution had a higher LnP(D) (−6006.6) than K = 2, but the individual ancestry patterns showed strong correspondence between the two solutions (r = 0.898). In particular, 42 of the 46 individuals predominantly assigned to K = 3 cluster 3 corresponded to K = 2 cluster 2, while K = 2 cluster 1 was mainly subdivided between K = 3 clusters 1 and 2. The proportion of individuals with Q ≥ 0.80 was similar and slightly lower at K = 3 (81/120; 67.5%) than at K = 2 (85/120; 70.8%). These results indicate that K = 3 represents a finer subdivision of the principal K = 2 structure rather than an additional independent evolutionary lineage. The data for K = 3 are reported in the same tables as those for k = 2, within the cited tables in the Supplementary Materials. Expected heterozygosity was 0.8401 for cluster 1, 0.6902 for cluster 2, and 0.8779 for cluster 3. These values are reported descriptively and were not subjected to an inferential statistical comparison, because the Structure output provides cluster-level estimates rather than independent replicate estimates suitable for significance testing.

Therefore, the observed differences in He should not be interpreted as statistically significant differences in genetic diversity among clusters. The Structure-derived FST estimates were interpreted as cluster-specific differentiation estimates and not as pairwise FST values between independent populations. The estimates were 0.0547 for cluster 1, 0.1167 for cluster 2, and 0.0003 for cluster 3 (Table S5).

Finally, the combination of LDH-C1* and microsatellite data provides a more informative picture of the genetic composition of Campania trout populations than either marker system considered independently (Table S6). The results indicate that the second cluster at K = 2 and the third cluster at K = 3 consist predominantly of individuals homozygous for the MED allele at the LDH-C1* locus and may therefore represent the groups most closely associated with the native Mediterranean brown trout genetic background. The remaining clusters include individuals showing evidence of admixture or introgression. It is important to point out that the presence of discrepancies between LDH-C1* classification and Structure assignment are not necessarily contradictory: probably they may reflect the different genomic scales represented by the two approaches, particularly in populations affected by historical introgression. The three LDH-C1* categories should be regarded as diagnostic genotype classes, whereas Structure clusters represent statistical components of multi locus genetic variation. In admixed populations, historical introgression may therefore produce individuals whose single *locus* genotype and multi *locus* ancestry do not correspond exactly as reported by Ayala et al. [49]. Similarly, mitochondrial *16S rRNA* marker provides information on maternal lineage and was analyzed only in specimens classified as MED by LDH-C1*. In contrast, multi *locus* microsatellites provide a broader assessment of biparental nuclear ancestry and can reveal admixture patterns not identified by a single *locus*.

Furthermore, similar discrepancies have been reported in trout populations, including the 52% concordance reported by Veličković et al. [50]. Previous studies have also highlighted the value of multi locus nuclear markers for improving lineage assignment and reducing potential misclassification in conservation programs [51,52].

## 4. Conclusions

This study provides an exploratory assessment of *Salmo trutta* populations inhabiting the main river basins of the Campania region. It highlights the limitations of preliminary morphological assessments and underscores the importance of integrating genetic analyses for accurate species identification and robust lineage delineation. The results have demonstrated the effectiveness of the genetic analysis to clarify hybridization dynamics and to support the conservation of lineage integrity, particularly in river systems increasingly influenced by restocking activities and environmental pressures [44,53,54,55,56]. Specifically, it is important to highlight that the differences between LDH-C1* genotyping and microsatellites data evidence the importance of adopting an integrative genetic approach to improve the accuracy of lineage identification for *Salmo trutta* populations in Campania as well as in other geographic area. For example, to preserve the evolutionary potential and ecological resilience of *Salmo trutta* in southern Italy, conservation programs should prioritize the genetic identification and protection of putatively native or least-introgressed Mediterranean populations, such as those of the Imperatore, Sava, and Volturno rivers.

Overall, the results suggest that restocking with predominantly Atlantic hatchery fish may have altered the genetic composition of local trout population, contributing to the erosion of native Mediterranean lineages. Nevertheless, the present study has some limitations, including the preliminary nature of morphological assessment and the lack of reliable information on the age and maturity stages of the wild specimens. Further studies, combining comprehensive morphometric and ecological characterization with multi *locus* or genomic approaches and larger sample sizes, will be essential to refine the assessment of population structure and Atlantic introgression.

In this context, conservation efforts should focus on establishing broodstocks of putatively native Mediterranean trout (confirmed through multi *locus* genetic analyses) and on implementing restocking programs based exclusively on native genetic material. Finally, the use of microsatellite and genomic markers could represent a valid approach for monitoring the effectiveness of these actions and safeguarding the long-term integrity of the native trout population in Campania.

## Figures and Tables

**Table 1:** Sampling stations (acronyms), locations, geographic coordinates, and number of collected trout specimens.

N°	Sampling Station ID	River Station	Locations	Geographic Coordinates		Number of Trout
				Latitude	Longitude	
1	V1	Volturno	Sava Mouth–Capriati al Volturno (CE)	41.489619°	14.112860°	26
2	T2a	Torano	Bridge Molino Vernelle–Alife (CE)	41.294743°	14.340396°	6
3	Sv1	Savone	Tuoro–Teano (CE)	41.269662°	14.041301°	25
4	Sa1	Sava	Molino–Capriati al Volturno (CE)	41.463604°	14.140557°	23
5	Lt1	Lete	Letino (CE)–Secine	41.447780°	14.304757°	30
6	Cl2	Calore Sal	Ancient Bridge–Laurino (SA)	40.336538°	15.329883°	6
7	Cl3bis	Calore Sal	Street Remolino–Felitto (SA)	40.366039°	15.251757°	3
8	S1	Sabato	Santo Stefano del Sole–Villa San Nicola	40.888288°	14.846605°	25
9	S2	Sabato	Atripalda–Serino road	40.905197°	14.837871°	19
10	S3	Sabato	Atripalda–bridge Milano	40.913020°	14.827745°	13
11	Ve1	Vellola	Altavilla Irpina	41.014512°	14.775766°	14
12	Pi1	Picentino	Nocelleto Infrattata–Giffoni Valle Piana	40.756707°	14.963162°	17
13	Pi2	Picentino	Giffoni Valle Piana	40.764528°	14.982722°	23
14	Pi3	Picentino	Vassi Giffoni Valle Piana	40.703500°	14.934071°	20
15	Pi	Picentino	Bridge Ornito–Giffoni Valle Piana	40.703500°	14.934071°	25
16	Pal1	Palistro	Massascusa–Ceraso (SA)	40.203479°	15.274331°	14
17	Pal1bis	Palistro	near the ichthyogenic center of Ceraso	40.198959°	15.300378°	25
18	Tn2	Tanagro	Petroso Stream– Sicignano d’Alburni (SA)	40.583611°	15.357361°	2
19	Tn3bis	Tanagro	Piana San Vito–Sicignano d’Alburni (SA)	40.610235°	15.255547°	13
20	Tn1ter	Tanagro	Acquariello–Montesano on the Marcellana (SA)	40,307948°	15.628217°	12
21	Imp1	Imperatore	Montesano on the Marcellana (SA)	40,273292°	15.676808°	30
22	Por1	Porcile	Montesano on the Marcellana (SA)	40.263004°	15.671272°	4
23	Tu1bis	Tusciano	torrent tramafiume–Acerno	40.723384°	15.035300°	30
24	Tu1ter	Tusciano	torrent tramafiume–Acerno	40.723654°	15.038091°	24
25	Tu1	Tusciano	Acerno	40.718972°	15.040432°	24
26	Tu2	Tusciano	Olevano S/T	40.644721°	15.012040°	23
27	Ten1	Tenza	Campagna	40.39598°	15.062297°	14
28	Sl3	Sele	Industrial area of Contursi Terme	40.637764°	15.233715°	18
29	Sl1	Sele	Calabritto–Materdomini	40.784540°	15.240140°	88

**Table 2:** Primer pairs, labels, and references for microsatellite genotyping.

Locus	Dye	Reapeat	Range (bp)	Primer F	Primer R	Multiplex	Reference
SssaD190	HEX	Tetra	115–151	5′-GGCATTGGAG GTAAGGACAC-3′	5′-CCAGACCACTGAA CTTCTCATC-3′	M1	[37]
SssaD71	FAM	Tetra	191–263	5′-AACGTGAAACA TAAATCGATGG-3′	5′-TTAAGAATGGGTTG CCTATGAG-3′	M1	[37]
SSsp2213	ROX	Tetra	151–227	5′-ATGTGGAGGTCAA CTAACCAGCGTTG-3′	5′-CATCAATCACAGA GTGAGGCAACTCG-3′	M1	[38]
Ssa85	FAM	Di	102–114	5′-AGGTGGGTCCTC CAAGCTAC-3′	5′-ACCCGCTCCTCAC TTAATC-3′	M2	[39]
Ssa410Uos	ROX	Tetra	126–250	5′-GGAAAATAATC AATGCTGCTGGTT-3′	5′-CTACAATCTGGA CTTCTTCTTCA-3′	M2	[40]
Str73INRA	TAMRA	Di	114–150	5′-CCTGGAGATCCTC CAGCAGGA-3′	5′-CTATTCTGCTTGT AACTAGACCTA-3′	M3	[41]
Str60INRA	FAM	Di	95–105	5′-CGGTGTGCTTGTC AGGTTTTC-3′	5′-GTCAAGTCAGCA AGCCTCAC-3′	M3	[41]
SssoSL417	ROX	Di	169–199	5′-TTGTTCAGTGTAT ATGTGTCCCAT-3′	5′-GATCTTCACTGC CACCTTATGACC-3′	M3	[42]
Sssa408Uos	HEX	Tetra	204–276	5′-AATGGATTACGG GTACGTTAGACA-3′	5′-CTCTTGTGCAGG TTCTTCATCTGT-3′	M3	[40]
Str15INRA	FAM	Di	180–186	5′-TGCAGGCAGACG GATCAGGC-3′	5′-AATCCTCTAACG TAAGGGATTTGC-3′	M4	[41]
Strutta58	HEX	Di	228–316	5′-AACAATGACTTT CTCTGAC-3′	5′-AAGGACTTGAA GGACGAC-3′	M4	[21]
T3–13	ROX	Di	189–261	5′-CCAGTTAGGGTT CATTGTCC-3′	5′-CGTTACACCT CTCAACAGATG-3′	M4	[41]

## Data Availability

The original contributions presented in this study are included in the article/Supplementary Materials. Further inquiries can be directed to the corresponding authors.

## Supplementary Materials

The following supporting information can be downloaded at: https://www.mdpi.com/article/10.3390/ani16193061/s1, **Table S1. Individual *LDH-C1** and mitochondrial *16S rRNA* genotypes of the selected trout specimens.** MED, Mediterranean-associated genotype; ATL, Atlantic-associated genotype; HYB, hybrid genotype; **Table S2. *Locus*-level quality-control of the microsatellite dataset**. N genotypes (number of successfully genotyped individuals); N Missing (number of missing genotypes); Missing % (percentage of missing genotypes); Alleles (number of observed alleles); He (expected heterozygosity); Ho (observed heterozygosity); Brookfield null allele estimate (estimated frequency of potential null allele based on heterozygote deficiency); **Table S3. Summary statistics from the Structure analyses at K = 2 and K = 3**. Overall membership expected heterozygosity and FST are reported for each inferred cluster. LnP(D), mean log likelihood, variance of log likelihood and alpha are run-level Structure statistics; **Table S4. Individual-level Structure membership coefficients for the 120 genotyped *Salmo trutta* specimens under K = 2 and K = 3**. Q-values represent the estimated proportion assigned to each inferred genetic component for each individual. Individuals with Q < 0.80 for all components are classified as admixed (<0.80); **Table S5. *Locus*-specific differentiation screening based on the K = 3 Structure assignments**. Values represent the estimated FST between the indicated inferred Structure components for each microsatellite *locus*; **Table S6. Distribution of *LDH-C1** genotype classes in the complete dataset and in the 120-individual microsatellite Structure subset**. HYB, hybrid/intermediate genotype; MED, Mediterranean-associated genotype; ATL, Atlantic-associated genotype.

## Abbreviations

The following abbreviations are used in this manuscript:

*S.*	*Salmo*
ATL	Atlantic
MED	Mediterranean
AD	Adriatic
DA	Danubian
MA	Marmoratus
AIIAD	Italian Association of Fresh Water Ichthyologists
ESU	Evolutionarily significant units
*LDH-C1**	*Lactate dehydrogenase-C1*
RFLP-PCR	Restriction Fragment Length Polymorphism—PCR
MCMC	Monte-Carlo Markov-Chain
HYB	Hybrids
