# Supplementary material for: An Exploratory Analysis of Genetic Structure and Introgression in Salmo trutta Populations from Campania Rivers
Source: Animals (Basel). 2026 Sep 29;16(19):3061. doi: 10.3390/ani16193061 (PMC13663513; doi:10.3390/ani16193061)
Supplement: Supplementary file 1 [file animals-16-03061-s001.zip › animals-4566461-supplementary.pdf]

Supplementary Materials

An exploratory analysis of genetic structure and introgression in *Salmo trutta* populations from Campania rivers.

Table S1. Individual *LDH-C1 and mitochondrial *16S rRNA* genotypes of the selected trout specimens.** MED, Mediterranean-associated genotype; ATL, Atlantic-associated genotype; HYB, hybrid genotype.

ID Samples	LDH-C1	16 S
SL3.4	MED	MED
SL3.6	MED	MED
SL3.12	MED	MED
SL3.13	MED	MED
SL3.14	MED	MED
SL3.15	MED	MED
TU1.4	MED	MED
TU1.6	MED	MED
TU1.16	MED	MED
TU1.17	MED	MED
TU1.19	MED	MED
TU1.20	MED	MED
TU1.21	MED	MED
TU1.22	MED	MED
TU2.4	MED	MED
TU2.16	MED	MED
TU2.17	MED	MED
TU2.18	MED	MED
TU2.19	MED	MED
IMP.3	MED	MED
IMP.4	MED	MED
IMP.5	MED	MED
IMP.6	MED	MED
IMP.7	MED	MED
IMP.8	MED	MED
IMP.10	MED	MED
IMP.11	MED	MED
IMP.12	MED	MED
IMP.13	MED	MED
IMP.14	MED	MED
IMP.15	MED	MED
IMP.17	MED	MED
IMP.18	MED	MED
IMP.19	MED	MED

IMP.20	MED	MED
IMP.22	MED	MED
IMP.23	MED	MED
IMP.24	MED	MED
IMP.26	MED	MED
IMP.27	MED	MED
IMP.28	MED	MED
IMP.30	MED	MED
TN1TER.1	MED	MED
TN1TER.3	MED	MED
TN1TER.10	MED	MED
SA1.1	MED	MED
SA1.2	MED	MED
SA1.4	MED	MED
SA1.5	MED	MED
SA1.6	MED	MED
SA1.7	MED	MED
SA1.9	MED	MED
SA1.10	MED	MED
SA1.11	MED	MED
SA1.12	MED	MED
SA1.13	MED	MED
SA1.14	MED	MED
SA1.15	MED	MED
SA1.16	MED	MED
SA1.17	MED	MED
SA1.18	MED	MED
SA1.19	MED	MED
SA1.20	MED	MED
SA1.21	MED	MED
SA1.22	MED	MED
Pi2.3	MED	MED
Pi2.4	MED	MED
Pi2.5	MED	MED
Pi2.6	MED	MED
Pi2.15	MED	MED
Pi2.16	MED	MED
Pi2.18	MED	MED
Pi2.21	MED	MED
Pi2.23	MED	MED
Pi1-1	MED	MED
Pi1-2	MED	MED
Pi1-4	MED	MED

Pi1-7	MED	MED
Pi1-8	MED	MED
Pi1-9	MED	MED
Pi1-11	MED	MED
Pi1-14	MED	MED
Pi1-15	MED	MED
V1-1	MED	MED
V1-2	MED	MED
V1-3	MED	MED
V1-4	MED	MED
V1-5	MED	MED
V1-6	MED	MED
V1-7	MED	MED
V1-8	MED	MED
V1-9	MED	MED
V1-11	MED	MED
V1-12	MED	MED
V1-13	MED	MED
V1-14	MED	MED
V1-15	MED	MED
V1-17	MED	MED
V1-18	MED	MED
V1-20	MED	MED
V1-21	MED	MED
V1-22	MED	MED
V1-23	MED	MED
V1-24	MED	MED
V1-25	MED	MED
LT1-3	MED	MED
LT1-4	MED	MED
LT1-5	MED	MED
LT1-6	MED	MED
LT1-10	MED	MED
LT1-14	MED	MED
LT1-15	MED	MED
LT1-16	MED	MED
LT1-18	MED	MED
LT1-26	MED	MED
TEN1.6	MED	MED
CL2.3	MED	MED
CL2.4	MED	MED
CL2.5	MED	MED
CL3BIS.1	MED	MED

CL3BIS.2	MED	MED
CL3BIS.3	MED	MED
SL1.2	MED	MED
SL1.35	MED	MED
SL1.36	MED	MED
SL1.39	MED	MED
SL1.40	MED	MED
SL1.42	MED	MED
SL1.44	MED	MED

Table S2. ***Locus*****-level quality-control of the microsatellite dataset.** N genotypes (number of successfully genotyped individuals); N Missing (number of missing genotypes); Missing % (percentage of missing genotypes); Alleles (number of observed alleles); He (expected heterozygosity); Ho (observed heterozygosity); Brookfield null allele estimate (estimated frequency of potential null allele based on heterozygote deficiency).

**Locus**	**N genotypes**	**N Missing**	**Missing %**	**Alleles**	**Expected heterozygosity (He)**	**Observed heterozygosity (Ho)**	**Brookfield null allele estimate**
**ssad71**	107	13	10,83333333	68	0,960389554	0,700934579	0,132348682
**ssad190**	105	15	12,5	54	0,914648526	0,676190476	0,124544033
**ssp2213**	108	12	10	67	0,923139575	0,694444444	0,118917594
**ssa85**	111	9	7,5	34	0,826962097	0,387387387	0,240604176
**str73inra**	110	10	8,333333333	14	0,726446281	0,054545455	0,389181427
**ssa410uos**	112	8	6,666666667	88	0,969786352	0,830357143	0,070783925
**strl60inra**	112	8	6,666666667	20	0,723453444	0,241071429	0,279892687
**ssa408uos**	110	10	8,333333333	81	0,967933884	0,836363636	0,066857047
**ss0sl417**	108	12	10	45	0,807570302	0,462962963	0,190646714
**str15inra**	118	2	1,666666667	36	0,708093939	0,254237288	0,26570942
**strutta58**	118	2	1,666666667	75	0,960535766	0,906779661	0,027419089
**t313**	117	3	2,5	74	0,957082329	0,897435897	0,030477221

Table S3. Summary statistics from the Structure analyses at K = 2 and K = 3. Overall membership expected heterozygosity and FST are reported for each inferred cluster. LnP(D), mean log likelihood, variance of log likelihood and alpha are run-level Structure statistics.

Solution	Metric	Cluster 1	Cluster 2	Cluster 3
K = 2	Overall membership	0.496	0.504	—
K = 2	Expected heterozygosity	0.7951	0.8743	—
K = 2	FST estimate	0.0555	0.0004	—
K = 2	LnP(D)	−6134.3	—	—
K = 2	Mean log likelihood	−5927.6	—	—
K = 2	Variance of log likelihood	413.2	—	—
K = 2	Alpha	0.1477	—	—
K = 2	Net nucleotide distance	0.0180	—	—
K = 3	Overall membership	0.330	0.262	0.408
K = 3	Expected heterozygosity	0.8401	0.6902	0.8779
K = 3	FST estimate	0.0547	0.1167	0.0003
K = 3	LnP(D)	−6006.6	—	—
K = 3	Mean log likelihood	−5722.2	—	—
K = 3	Variance of log likelihood	568.8	—	—
K = 3	Alpha	0.1012	—	—

Table S4. Individual-level Structure membership coefficients for the 120 genotyped *Salmo trutta* specimens under K = 2 and K = 3. Q-values represent the estimated proportion assigned to each inferred genetic component for each individual. Individuals with Q < 0.80 for all components are classified as admixed (<0.80).

STRUCTURE individual ID	Sampling site	LDH-C1*	K2-Q1	K2-Q2	K2 assignment	K3-Q1	K3-Q2	K3-Q3	K3 assignment
**LT4**	Lete	MED	0,165	0,835	Cluster 2	0,232	0,035	0,733	Admixed (<0.80)
**SA113**	Sava	MED	0,025	0,975	Cluster 2	0,017	0,017	0,966	Cluster 3
**IMP14**	Imperatore	MED	0,055	0,945	Cluster 2	0,043	0,033	0,924	Cluster 3
**IMP7**	Imperatore	MED	0,038	0,962	Cluster 2	0,021	0,017	0,962	Cluster 3
**SA14**	Sava	MED	0,022	0,978	Cluster 2	0,014	0,015	0,971	Cluster 3
**SA17**	Sava	MED	0,018	0,982	Cluster 2	0,012	0,013	0,975	Cluster 3
**Pi223**	Picentino	MED	0,023	0,977	Cluster 2	0,018	0,014	0,968	Cluster 3
**SL314**	Sele	MED	0,191	0,809	Cluster 2	0,016	0,533	0,45	Admixed (<0.80)
**LT5**	Lete	MED	0,182	0,818	Cluster 2	0,197	0,079	0,724	Admixed (<0.80)
**Tn1TEN**	Tanagro	MED	0,035	0,965	Cluster 2	0,027	0,014	0,959	Cluster 3
**V19**	Volturno	MED	0,027	0,973	Cluster 2	0,023	0,016	0,961	Cluster 3
**SL40**	Sele	MED	0,065	0,935	Cluster 2	0,018	0,142	0,841	Cluster 3
**IMP18**	Imperatore	MED	0,015	0,985	Cluster 2	0,011	0,01	0,979	Cluster 3
**SA121**	Sava	MED	0,032	0,968	Cluster 2	0,014	0,035	0,951	Cluster 3
**IMP27**	Imperatore	MED	0,03	0,97	Cluster 2	0,023	0,013	0,964	Cluster 3
**P124**	Palistro	MED	0,053	0,947	Cluster 2	0,124	0,013	0,864	Cluster 3
**Pi221**	Picentino	MED	0,089	0,911	Cluster 2	0,148	0,024	0,829	Cluster 3
**V12**	Volturno	MED	0,026	0,974	Cluster 2	0,022	0,016	0,962	Cluster 3
**V123**	Volturno	MED	0,047	0,953	Cluster 2	0,016	0,052	0,932	Cluster 3
**V125**	Volturno	MED	0,02	0,98	Cluster 2	0,023	0,013	0,964	Cluster 3
**VI24**	Vellola	MED	0,066	0,934	Cluster 2	0,05	0,036	0,914	Cluster 3
**VI8**	Vellola	MED	0,014	0,986	Cluster 2	0,011	0,01	0,979	Cluster 3
**IMP4**	Imperatore	MED	0,021	0,979	Cluster 2	0,016	0,014	0,97	Cluster 3
**IMP26**	Imperatore	MED	0,027	0,973	Cluster 2	0,032	0,014	0,953	Cluster 3
**V1**	Volturno	MED	0,025	0,975	Cluster 2	0,067	0,014	0,919	Cluster 3
**CL3BIS2**	Calore	MED	0,422	0,578	Admixed (<0.80)	0,436	0,055	0,509	Admixed (<0.80)
**SA12**	Sava	MED	0,319	0,681	Admixed (<0.80)	0,534	0,116	0,35	Admixed (<0.80)
**TU16**	Tusciano	MED	0,922	0,078	Cluster 1	0,027	0,944	0,029	Cluster 2
**V13**	Volturno	MED	0,068	0,932	Cluster 2	0,62	0,02	0,36	Admixed (<0.80)
**PI1**	Picentino	MED	0,334	0,666	Admixed (<0.80)	0,847	0,04	0,113	Cluster 1
**TEN6**	Tenna	MED	0,87	0,13	Cluster 1	0,903	0,043	0,054	Cluster 1
**SA19**	Sava	MED	0,846	0,154	Cluster 1	0,564	0,218	0,219	Admixed (<0.80)
**IMP24**	Imperatore	MED	0,793	0,207	Admixed (<0.80)	0,813	0,045	0,141	Cluster 1
**V16**	Volturno	MED	0,365	0,635	Admixed (<0.80)	0,532	0,064	0,404	Admixed (<0.80)
**VI17**	Vellola	MED	0,349	0,651	Admixed (<0.80)	0,693	0,121	0,185	Admixed (<0.80)
**SL44**	Sele	MED	0,029	0,971	Cluster 2	0,027	0,017	0,957	Cluster 3
**LT14**	Lete	MED	0,608	0,392	Admixed (<0.80)	0,531	0,237	0,231	Admixed (<0.80)
**V112**	Volturno	MED	0,117	0,883	Cluster 2	0,713	0,019	0,268	Admixed (<0.80)
**IMP15**	Imperatore	MED	0,64	0,36	Admixed (<0.80)	0,413	0,08	0,507	Admixed (<0.80)
**IMP8**	Imperatore	MED	0,844	0,156	Cluster 1	0,236	0,347	0,416	Admixed (<0.80)

**LT3**	Lete	MED	0,142	0,858	Cluster 2	0,123	0,112	0,765	Admixed (<0.80)
**IMP20**	Imperatore	MED	0,584	0,416	Admixed (<0.80)	0,149	0,141	0,71	Admixed (<0.80)
**V113**	Volturno	MED	0,456	0,544	Admixed (<0.80)	0,896	0,031	0,073	Cluster 1
**SA116**	Sava	MED	0,079	0,921	Cluster 2	0,685	0,018	0,296	Admixed (<0.80)
**V17**	Volturno	MED	0,478	0,522	Admixed (<0.80)	0,434	0,301	0,265	Admixed (<0.80)
**PI216**	Picentino	MED	0,843	0,157	Cluster 1	0,698	0,083	0,219	Admixed (<0.80)
**SA115**	Sava	MED	0,589	0,411	Admixed (<0.80)	0,746	0,102	0,152	Admixed (<0.80)
**TUL17**	Tusciano	MED	0,751	0,249	Admixed (<0.80)	0,069	0,777	0,155	Admixed (<0.80)
**LT10**	Lete	MED	0,046	0,954	Cluster 2	0,119	0,008	0,873	Cluster 3
**SA118**	Sava	MED	0,036	0,964	Cluster 2	0,022	0,026	0,952	Cluster 3
**SL136**	Sele	MED	0,064	0,936	Cluster 2	0,02	0,117	0,863	Cluster 3
**V122**	Volturno	MED	0,012	0,988	Cluster 2	0,011	0,008	0,981	Cluster 3
**V112**	Volturno	MED	0,105	0,895	Cluster 2	0,062	0,061	0,876	Cluster 3
**PI215**	Picentino	MED	0,04	0,96	Cluster 2	0,029	0,028	0,943	Cluster 3
**LT126**	Lete	MED	0,037	0,963	Cluster 2	0,023	0,023	0,954	Cluster 3
**PI9**	Picentino	MED	0,621	0,379	Admixed (<0.80)	0,292	0,244	0,465	Admixed (<0.80)
**SA122**	Sava	MED	0,063	0,937	Cluster 2	0,36	0,026	0,614	Admixed (<0.80)
**TNTEN10**	Tanagro	MED	0,704	0,296	Admixed (<0.80)	0,564	0,065	0,372	Admixed (<0.80)
**TU22**	Tusciano	MED	0,276	0,724	Admixed (<0.80)	0,023	0,499	0,478	Admixed (<0.80)
**LT16**	Lete	MED	0,038	0,962	Cluster 2	0,062	0,021	0,917	Cluster 3
**PI23**	Picentino	MED	0,038	0,962	Cluster 2	0,08	0,014	0,905	Cluster 3
**IMP13**	Imperatore	MED	0,04	0,96	Cluster 2	0,032	0,015	0,952	Cluster 3
**SA117**	Sava	MED	0,056	0,944	Cluster 2	0,257	0,025	0,718	Admixed (<0.80)
**V15**	Volturno	MED	0,054	0,946	Cluster 2	0,069	0,037	0,894	Cluster 3
**TU9**	Tusciano	MED	0,96	0,04	Cluster 1	0,075	0,886	0,039	Cluster 2
**IMP6**	Imperatore	MED	0,869	0,131	Cluster 1	0,92	0,032	0,048	Cluster 1
**TU16**	Tusciano	MED	0,94	0,06	Cluster 1	0,063	0,875	0,062	Cluster 2
**TN1TEN3**	Tanagro	MED	0,937	0,063	Cluster 1	0,523	0,362	0,114	Admixed (<0.80)
**CL24**	Calore	MED	0,207	0,793	Admixed (<0.80)	0,316	0,059	0,625	Admixed (<0.80)
**SL315**	Sele	ATL	0,983	0,017	Cluster 1	0,075	0,908	0,017	Cluster 2
**TU24**	Tusciano	ATL	0,962	0,038	Cluster 1	0,095	0,871	0,034	Cluster 2
**IMP12**	Imperatore	ATL	0,088	0,912	Cluster 2	0,142	0,017	0,841	Cluster 3
**S22**	Sabato	ATL	0,786	0,214	Admixed (<0.80)	0,898	0,035	0,067	Cluster 1
**SL129**	Sele	ATL	0,786	0,214	Admixed (<0.80)	0,049	0,874	0,077	Cluster 2
**SL115**	Sele	ATL	0,969	0,031	Cluster 1	0,074	0,899	0,027	Cluster 2
**POR2**	Porcile	ATL	0,814	0,186	Cluster 1	0,923	0,023	0,054	Cluster 1
**SL62**	Sele	ATL	0,957	0,043	Cluster 1	0,026	0,95	0,024	Cluster 2
**IMP29**	Imperatore	ATL	0,932	0,068	Cluster 1	0,618	0,219	0,163	Admixed (<0.80)
**TU7TER**	Tusciano	ATL	0,971	0,029	Cluster 1	0,13	0,839	0,03	Cluster 2
**P316**	Palistro	ATL	0,776	0,224	Admixed (<0.80)	0,53	0,114	0,356	Admixed (<0.80)
**SL53**	Sele	ATL	0,28	0,72	Admixed (<0.80)	0,021	0,498	0,482	Admixed (<0.80)
**VEL5**	Velolla	ATL	0,934	0,066	Cluster 1	0,947	0,018	0,035	Cluster 1
**SL45**	Sele	ATL	0,513	0,487	Admixed (<0.80)	0,067	0,54	0,393	Admixed (<0.80)
**SL70**	Sele	ATL	0,913	0,087	Cluster 1	0,063	0,858	0,079	Cluster 2
**SL17**	Sele	ATL	0,9	0,1	Cluster 1	0,933	0,038	0,029	Cluster 1
**VEL8**	Velolla	ATL	0,977	0,023	Cluster 1	0,977	0,011	0,012	Cluster 1
**TU12BU**	Tusciano	ATL	0,817	0,183	Cluster 1	0,036	0,848	0,117	Cluster 2

**SL43**	Sele	ATL	0,787	0,213	Admixed (<0.80)	0,265	0,439	0,296	Admixed (<0.80)
**S2**	Sabato	ATL	0,721	0,279	Admixed (<0.80)	0,93	0,042	0,028	Cluster 1
**TNTEN6**	Tanagro	ATL	0,84	0,16	Cluster 1	0,57	0,125	0,306	Admixed (<0.80)
**S4**	Sabato	ATL	0,678	0,322	Admixed (<0.80)	0,961	0,013	0,026	Cluster 1
**S7**	Sabato	ATL	0,647	0,353	Admixed (<0.80)	0,954	0,022	0,025	Cluster 1
**TU20TER**	Tusciano	ATL	0,969	0,031	Cluster 1	0,089	0,886	0,025	Cluster 2
**TU3TER**	Tusciano	ATL	0,967	0,033	Cluster 1	0,052	0,924	0,023	Cluster 2
**TEN4**	Tenna	IBR	0,601	0,399	Admixed (<0.80)	0,77	0,051	0,179	Admixed (<0.80)
**TU17TER**	Tusciano	IBR	0,982	0,018	Cluster 1	0,011	0,979	0,01	Cluster 2
**SL65**	Sele	IBR	0,941	0,059	Cluster 1	0,047	0,9	0,053	Cluster 2
**SL116**	Sele	IBR	0,908	0,092	Cluster 1	0,024	0,937	0,038	Cluster 2
**TEN13**	Tenna	IBR	0,162	0,838	Cluster 2	0,768	0,019	0,214	Admixed (<0.80)
**PI11**	Picentino	IBR	0,954	0,046	Cluster 1	0,268	0,661	0,07	Admixed (<0.80)
**P29**	Palistro		0,382	0,618	Admixed (<0.80)	0,901	0,018	0,082	Cluster 1
**S12**	Sabato	IBR	0,35	0,65	Admixed (<0.80)	0,892	0,013	0,095	Cluster 1
**S23**	Sabato	IBR	0,842	0,158	Cluster 1	0,816	0,112	0,072	Cluster 1
**SL118**	Sele	IBR	0,889	0,111	Cluster 1	0,016	0,95	0,034	Cluster 2
**SL119**	Sele	IBR	0,58	0,42	Admixed (<0.80)	0,026	0,771	0,203	Admixed (<0.80)
**T2A2**	Torano	IBR	0,763	0,237	Admixed (<0.80)	0,784	0,121	0,095	Admixed (<0.80)
**SL11**	Sele	IBR	0,972	0,028	Cluster 1	0,018	0,967	0,016	Cluster 2
**SL73**	Sele	IBR	0,704	0,296	Admixed (<0.80)	0,277	0,581	0,142	Admixed (<0.80)
**S6**	Sabato	IBR	0,727	0,273	Admixed (<0.80)	0,962	0,015	0,023	Cluster 1
**TU19TER**	Tusciano	IBR	0,979	0,021	Cluster 1	0,033	0,951	0,016	Cluster 2
**T2A5**	Torano	IBR	0,975	0,025	Cluster 1	0,914	0,07	0,017	Cluster 1
**VEL7**	Velolla	IBR	0,976	0,024	Cluster 1	0,972	0,016	0,012	Cluster 1
**SL84**	Sele	IBR	0,944	0,056	Cluster 1	0,022	0,947	0,032	Cluster 2
**SL1187**	Sele		0,934	0,066	Cluster 1	0,025	0,938	0,038	Cluster 2
**TU12TER**	Tusciano	IBR	0,97	0,03	Cluster 1	0,034	0,942	0,025	Cluster 2
**PAL12BIS**	Palistro	IBR	0,861	0,139	Cluster 1	0,967	0,014	0,019	Cluster 1
**TNTER11**	Tanagro	IBR	0,966	0,034	Cluster 1	0,923	0,05	0,027	Cluster 1
**TU1BIS29**	Tusciano	IBR	0,712	0,288	Admixed (<0.80)	0,025	0,825	0,15	Cluster 2
**SL14**	Sele	IBR	0,628	0,372	Admixed (<0.80)	0,958	0,011	0,031	Cluster 1
**VEL11**	Velolla	IBR	0,927	0,073	Cluster 1	0,974	0,01	0,016	Cluster 1

Table S5. ***Locus*****-specific differentiation screening based on the K = 3 Structure assignments**. Values represent the estimated FST between the indicated inferred Structure components for each microsatellite *locus*.

Locus	K3 Cluster 1 vs 2	K3 Cluster 1 vs 3	K3 Cluster 2 vs 3
**SsaD71**	0,039	0,019	0,043
**SsaD190**	0,035	0,015	0,037
**SSsp2213**	0,152	0,03	0,115
**Ssa85**	0,137	0,044	0,109
**Str73INRA**	0,074	0,109	0,18
**Ssa410Uos**	0,017	0,016	0,016
**Str60INRA**	0,076	0,076	0,173
**Ssa408Uos**	0,037	0,019	0,034
**SsoSL417**	0,144	0,023	0,176
**Str15INRA**	0,06	0,06	0,122
**Strutta58**	0,015	0,015	0,025
**T3-13**	0,023	0,012	0,028

Table S6. Distribution of *LDH-C1 genotype classes in the complete dataset and in the 120-individual microsatellite Structure subset.** HYB, hybrid/intermediate genotype; MED, Mediterranean-associated genotype; ATL, Atlantic-associated genotype.

Dataset	N	HYB	MED	ATL
All sampled specimens	596	387	129	80
Structure subset	120	16	73	31
